# Electrophoretic Deposition of Aged and Charge Controlled Colloidal Copper Sulfide Nanoparticles

**DOI:** 10.3390/nano11010133

**Published:** 2021-01-08

**Authors:** Yoonsu Park, Hyeri Kang, Wooseok Jeong, Hyungbin Son, Don-Hyung Ha

**Affiliations:** School of Integrative Engineering, Chung-Ang University, 84 Heukseok-ro, Dongjak-gu, Seoul 06974, Korea; -ung2150@cau.ac.kr (Y.P.); rkdgofl96@cau.ac.kr (H.K.); woozreal@cau.ac.kr (W.J.)

**Keywords:** electrophoretic deposition, colloidal nanoparticle, nanostructured film, copper sulfide

## Abstract

Colloidal nanoparticles (NPs) have been recently spotlighted as building blocks for various nanostructured devices. Their collective properties have been exhibited by arranging them on a substrate to form assembled NPs. In particular, electrophoretic deposition (EPD) is an emerging fabrication method for such nanostructured films. To maximize the benefits of this method, further studies are required to fully elucidate the key parameters that influence the NP deposition. Herein, two key parameters are examined, namely: (i) the aging of colloidal NPs and (ii) the charge formation by surface ligands. The aging of Cu_2-x_S NPs changes the charge states, thus leading to different NP deposition behaviors. The SEM images of NP films, dynamic light scattering, and zeta potential results demonstrated that the charge control and restoration of interparticle interactions for aged NPs were achieved via simple ligand engineering. The charge control of colloidal NPs was found to be more dominant than the influence of aging, which can alter the surface charges of the NPs. The present results thus reveal that the charge formation on the colloidal NPs, which depends on the surface ligands, is an important controllable parameter in EPD.

## 1. Introduction

Colloidal nanoparticles (NPs) have been highlighted as functionalized nanomaterials in various research fields [1,2]. Numerous synthesis approaches have been reported for colloidal NPs, with various compositions and morphologies, including nanodots, nanorods, nanotubes, and nanosheets [3,4]. These colloidal syntheses have great advantages in achieving narrow size distributions with various specific sizes, which can control the shape- and size-dependent properties of NPs [5,6,7]. After synthesis, the colloidal NPs must be arranged on a substrate as building blocks to access unique properties and realize nanostructured devices [8,9,10]. Several solution-based NP film fabrication methods are available, including drop-casting, dip-casting, spin-coating, spray-coating, and injection printing. In addition, the process of electrophoretic deposition (EPD) is an emerging technique for the fabrication of NP films from colloidal NPs [9,11,12,13,14]. This process has various advantages, such as strong adhesion of NPs to the substrate and a wide range of applicable materials for both NPs and substrates. Depending primarily upon their surface charges, colloidal NPs dispersed in non-polar solvents (e.g., hexane and chloroform) or polar solvents (e.g., acetone and ethanol) can be selectively deposited onto either the positively- or negatively-charged substrate by the electric field formed during the EPD process. The deposition of NPs onto conductive substrates has been controlled via the tuning of process parameters, such as solvent, concentration, deposition time, and deposition voltage [15]. In particular, the stabilization of colloidal NPs by organic ligands (e.g., oleylamine and oleic acid) in non-polar solvent facilitates the control of the EPD process via limited current flow and suppressed electrochemical reactions at the electrodes compared to EPD of NPs in a polar solvent [16].

Colloidal copper sulfide (Cu_2-x_S) NPs are considered ideal for investigating the EPD mechanism in a non-polar solvent because they can adopt a variety of sizes/shapes and can be well dispersed in hexane or chloroform. Depending upon the stoichiometry, Cu_2-x_S (0 ≤ x ≤ 1) has various phases ranging from Cu-rich chalcocite (Cu_2_S) to covellite (CuS) [17]. The Cu_2-x_S phases with copper vacancies in the atomic structure are promising p-type semiconductors with unique optoelectrical properties due to their stoichiometry-dependent band gap [18]. In addition, localized surface plasmon resonance (LSPR) effects are exhibited in Cu_2-x_S NP due to the high free carrier density, depending on stoichiometry and size [19,20]. Hence, Cu_2-x_S NPs are suitable for nanostructured applications such as battery electrodes [21], photocatalysts [22], sensors [23], biomedical devices [24], and photovoltaic cells [25], as they provide unique properties that cannot be achieved using the traditional bulk materials. For example, Otelaja et al. (2014) [26] demonstrated that the EPD films obtained from Cu_2-x_S NPs dispersed in hexane exhibit a higher conductivity than those prepared by spin-casting. Further, Ha et al. (2015) [27] used a non-polar solvent-based additive-free EPD process to fabricate Cu_2-x_S NP Li-ion battery electrodes with high capacity and cyclability, and a strong adherence to the substrate. However, further studies are required to fully understand the key EPD parameters that control the deposition mechanism and, thus, maximize the benefits of the EPD process for colloidal NPs.

In non-polar solvents, the surface charge on colloidal NPs is mainly influenced by the ligand, which serves to generate repulsive interactions or electrostatic attractions between the colloidal NPs [16]. Thus, to control the NP deposition mechanism during the EPD process, an understanding of the parameters associated with charge formation on NPs is required [28]. The altered charge of colloidal NPs dispersed in a non-polar solvent can be simply adjusted by modulating the ligand coverage on the NP surface via additional precipitation steps or the addition of excess ligand [29,30]. A change in ligand coverage via the partial removal or additional adsorption of the ligands has significant influence upon the direction of particle movement between both electrodes and upon the morphology of the NP assembly formed on a substrate via EPD [28,31]. Although such ligand engineering approaches have been applied to simple systems for selective deposition, the mechanism and controllability are not yet well established.

In this work, the aging process and surface ligand concentration of colloidal NPs are demonstrated as key EPD parameters influencing the NP deposition mechanism. The synthesized Cu_2-x_S NPs dispersed in hexane are shown to exhibit partial oxidation through an aging process under the air atmosphere, thus leading to altered charge states of NPs and a reduction in the NP film uniformity compared to that generated using non-aged NPs. For implementing controllable deposition behavior during the EPD process, the ligands were shown to play a major role in controlling the surface charge on the colloidal NPs and stabilizing the interparticle interactions in a non-polar solvent such as hexane. A simple ligand engineering process was used to modulate the influence of aging on the NP charge, and the recovered NPs were found to display an altered deposition behavior by moving in the opposite direction compared to that of the non-aged NPs during the EPD process. This effect suggests that the concentration of surface ligands on the colloidal Cu_2-x_S NPs plays a crucial role in charge formation, having an even more significant impact than the surface oxidation process. In addition, a uniform Cu_2-x_S NP film with morphology similar to that of the non-aged NP film is successfully fabricated using the recovered NPs. The present work provides insights for understanding the mechanism of the EPD process with colloidal NPs and for realizing the fabrication of devices based on Cu_2-x_S NPs.

## 2. Materials and Methods

### 2.1. Chemicals and Materials

Oleylamine (OLAM, 70%, technical grade), di(tertbutyl) disulfide (TBDS, 97%), and copper (II) chloride dihydrate (CuCl_2_∙2H_2_O, ≥99%, ACS reagent) were purchased from Sigma-Aldrich. Acetone (C_3_H_6_O, 99.5%, extra pure) and n-hexane (CH_3_(CH_2_)_4_CH_3_, 95%, extra pure) were purchased from Daejung Chemicals & Metals. All chemicals were used without further purification.

### 2.2. Synthesis of Colloidal Cu_2-x_S Nanoparticles

The Cu_2-x_S NPs were synthesized via a slightly modified form of the standard procedure [32]. Standard Schlenk line techniques were used under a nitrogen atmosphere or vacuum. OLAM (30 mL) and CuCl_2_∙2H_2_O (1.704 g) were loaded into a 50 mL three-necked flask, equipped with an evaporator trap, thermometer, thermometer adapter, rubber septum, and magnetic stir bar. The solution was stirred under vacuum for 20 min at room temperature followed by an additional 1 h at 120 °C to remove impurities such as water and volatile chemicals. The solution was then maintained under a N_2_ atmosphere and heated to 200 °C for 1 h until the solution turned to a transparent yellow-green color. The solution was then cooled to 180 °C prior to injection of the sulfur source. The TBDS solution (4 mL) was then quickly injected by syringe into the flask, and the reaction progressed for 1 h. After the reaction, the heating mantel was removed, and the reaction vessel was quenched in a water bath. NPs were then collected by centrifugation and washed 3 times with hexane/acetone (≈1:3 *v*/*v*) at 5000 rpm for 5 min. Finally, the Cu_2-x_S NPs were dispersed in hexene and stored in a clear vial under an atmosphere of air.

### 2.3. Preparation of Nanoparticle Film via the EPD Process

**Figure 1:**
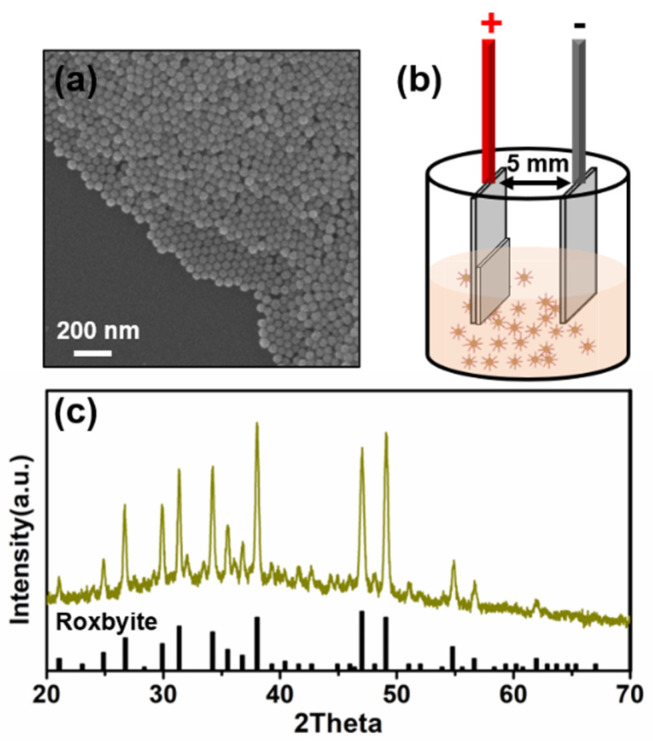
Characterization and electrophoretic deposition (EPD) of the fabricated Cu_2-x_S nanoparticles (NPs): (**a**) SEM image; (**b**) schematic EPD set-up; and (**c**) XRD pattern, with the black bars underneath corresponding to the reference data for the roxbyite phase (JCPDS #23-0958).

Silicon (Si) wafers (P type, the thickness of 525 μm, the dimensions of 1.5 × 1.5 cm^2^) were attached to a pair of stainless-steel plates, and the distance between the electrodes was adjusted to 5 mm. The substrate was cleaned in acetone with ultrasonication. The previously stored NPs were removed from the vial and dissolved in hexane in a tall beaker to a concentration of 0.59 g/L. The pair of electrodes were inserted into the resulting solution and a DC voltage (≈500 V) was applied and maintained for a deposition time of 2 min, as shown schematically in Figure 1b. During this stage, the charged colloidal NPs were attracted to the oppositely charged electrode, and the dark brown color of the solution gradually became light brown as the concentration of NPs in the solution decreased with deposition. The dried NP-coated substrate was then detached from the electrode, and analysis was performed to determine the surface morphology and chemical characteristics of the NP film.

### 2.4. Preparation and Recovery of Aged Nanoparticles

To investigate the influence of Cu_2-x_S NP aging upon the deposition mechanism, films were prepared on both positively and negatively charged substrates using non-aged NPs and NPs with different aging periods (i.e., 72, 192, 312, 528, and 720 h). For the recovery process, OLAM (9 mmol) was added to a dispersion of the aged Cu_2-x_S NPs (≈5.9 mg) in hexane (5 mL), and sonication was performed for 15 min. The solution was then washed by the addition of hexane/acetone (≈1:3 *v*/*v*) and centrifuged for 5 min at 5000 rpm. The resulting precipitate was completely dried and then re-dispersed in hexane by sonication. The recovered NP solution was immediately used in the EPD process to avoid any effects due to further aging.

### 2.5. Materials Characterization

The NP films prepared on Si wafers via the EPD process were characterized as follows. The X-ray diffraction (XRD) patterns were collected using an AXS New D8 advance diffractometer (Bruker, Billerica, MA, USA) with a Cu K-α radiation source and a LynxEye line detector. The samples for XRD were prepared by drop-casting the Cu_2-x_S NPs onto zero-background quartz. Field-emission scanning electron microscopy (FE-SEM) and energy-dispersive spectrometry (EDS) were performed using a Carl Zeiss SIGMA microscope. In addition, X-ray photoelectron spectroscopy (XPS) was performed on a K-alpha system (Thermo Fisher Scientific, Waltham, MA, USA) with an Al K-α source. FTIR spectroscopy was performed in a Nicolet 6700 spectrometer (Thermo Fisher Scientific) at room temperature. The FTIR samples were prepared as a powder. Zeta potential and dynamic light scattering (DLS) measurements were performed using a Malvern Zetasizer Pro (Malvern Instruments, Malvern, WR, UK) instrument with a universal dip cell kit for samples in non-aqueous (palladium electrodes with 2 mm spacing).

## 3. Results and Discussion

The SEM image of the synthesized Cu_2-x_S NPs in Figure 1a reveals a quasi-tetradecahedron shape, with a uniform particle size of ≈40 nm, and the XRD pattern in Figure 1c is well matched with the roxbyite reference (JCPDS #23-0958) [27]. As detailed in Section 2.3, these roxbyite NPs were dispersed in hexane and used in the NP film fabrication via the EPD process (Figure 1b). To maximize the morphological properties of the fabricated films (which should be crack-free, with high uniformity and density), the inherent properties of the colloidal NPs, such as dispersibility and size distribution, should first be optimized [33,34]. However, aggregation or oxidation of the NPs may occur during aging in solution [35,36,37,38,39]. This aging phenomenon influences both the inherent properties of the materials and the arrangement of the assembled NPs by altering the NP–NP or NP–substrate interactions. In the present study, the colloidal Cu_2-x_S NPs displayed a significant decrease in dispersibility in hexane due to the process of aging in the atmosphere of air, possibly due to considerable agglomeration of the particles via the altered interparticle interaction.

**Figure 2:**
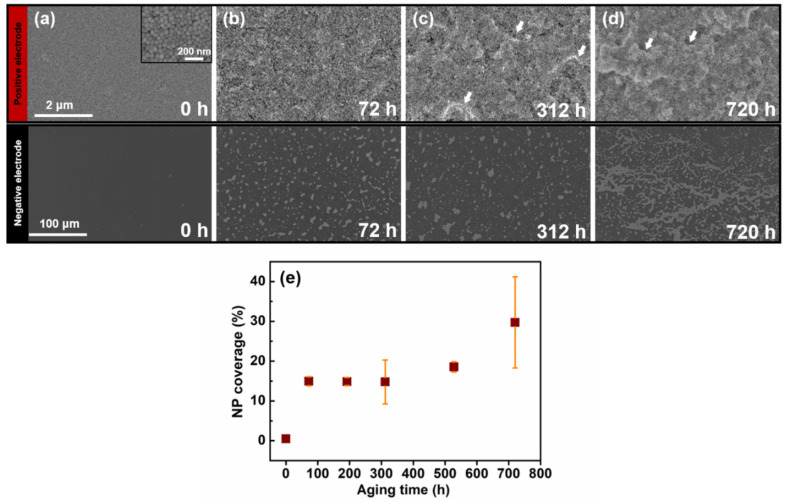
SEM images (**a**–**d**) of the films fabricated from Cu_2-x_S NPs after various aging times (top panel: positive electrode; bottom panel: negative electrode). For the negative electrode, a plot of NP surface coverage against aging time is presented in (**e**).

To investigate the influence of Cu_2-x_S NP aging upon the deposition mechanism, NP films were prepared using non-aged and variously-aged NPs, and the surface morphologies were analyzed as described in Section 2.4. The surface morphologies of the NP films obtained on the positively and negatively charged substrates are indicated by the SEM images in Figure 2 (more images in Figures S1 and S2 of the Supplementary Material). The NP film fabricated using non-aged NPs were formed mainly on the positively charged substrate (Figure 2a, upper panel), with few NPs being deposited on the negatively charged substrate (Figure 2a, lower panel). Moreover, the NP film on the positively charged substrate exhibited a uniform and dense surface (inset, Figure 2a). These results indicate that the net charge on the non-aged NPs is negative and that the interactions between the NPs are balanced. By comparison, the films prepared using the 72 h-aged NPs maintained a high density on the positively charged substrate, but the surface uniformity was decreased (Figure 2b, upper panel) compared to that of the non-aged NPs. In addition, several 72 h-aged NP domains were clearly visible on the negatively charged substrate (Figure 2b, lower panel). Meanwhile, the film formed on the positively charged substrate using the 312 h-aged NPs exhibited poor surface uniformity, with locally pitted or raised areas indicated by the arrows in Figure 2c (upper panel). Moreover, several 312 h-aged NP domains were observed on the negatively charged substrate (Figure 2c, lower panel), and these display a larger size distribution than those obtained from the 72 h-aged sample.

The creation of assembled NP domains might be due to strong attractive interactions between NPs at the electrophoretic double layer near the electrode surface during the deposition process [40]. This can occur because the two-dimensional physical mobility on the electrode surface predominates over the irreversible adsorption of NPs drawn from the solution to the electrode surface by the electric field [41]. The SEM image of the film prepared from the 720 h-aged NPs (Figure 2d) revealed a high surface roughness, along with a similar morphology to that of the films obtained using 192 h-, 321 h-, and 528 h-aged NPs. This increase in surface roughness and decrease in surface uniformity of the films with increased pre-aging of the NPs was primarily attributed to the agglomeration of NPs during the aging process. In addition, the aging process alters the charge states of NPs, leading to changes in both the amount of deposited NPs and the size and number of NP domains on each type of electrode during the EPD process. The number of NP domains on the negatively charged substrate dramatically increased with the use of 720 h-aged NPs (Figure 2d) compared to all other samples. The increase in the number of particles deposited on the negatively charged substrate with increased aging time is indicated in terms of the percent-coverage of NP domains on the Si wafer in Figure 2e. Here, the change in NP deposition behavior with increased aging occurs in two distinct stages, with an initial sharp increase from 0.22% coverage by the non-aged (0 h) NPs to 14.94% coverage by the 72 h-aged NPs, followed by a gradual increase to ≈30% coverage by the 720 h-aged NPs. The results indicate that the initial aging process plays an important role in altering the surface charge of the Cu_2-x_S NPs, while the NP deposition behavior change after long-term (–720 h-) aging indicates that the net charge on the NPs becomes significantly more positive at this stage.

**Figure 3:**
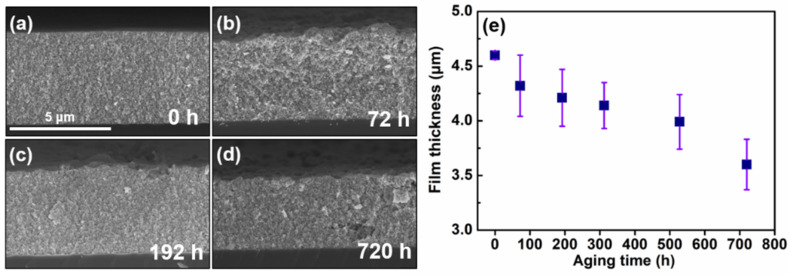
Cross-sectional SEM images (**a**–**d**) and dependence of film-thickness against pre-aging time (**e**) for films deposited on the positively charged substrate using variously-aged NPs.

The cross-sectional SEM images of some of the NP films deposited on the positively charged substrates are presented in Figure 3, while cross-sectional SEM images of all the positive-substrate samples are provided in Figure S3. In addition, a plot of film thickness against NP pre-aging time is provided (Figure 3e). The film thickness continuously decreases with increased NP pre-aging, although there is a high standard deviation for all films, except that produced using the non-aged NPs. The sample obtained from the non-aged NPs displays a smooth surface with uniform thickness of 4.6 μm (Figure 3a,e), while the 72 h-aged NPs result in a slightly decreased thickness of 4.3 ± 0.28 μm (Figure 3b,e). This corresponds to the first stage of altered deposition behavior noted above, in which the number of NPs attached to the negatively charged substrate increases significantly for the 72 h-aged NPs, compared to the non-aged NPs. By comparison, the NP film produced from the 192 h-aged NPs exhibits a slight further decrease in thickness to 4.2 ± 0.26 µm (Figure 3c,e), while the 720 h-aged NPs provide a significant additional decrease to 3.6 ± 0.23 µm (Figure 3d,e), along with a much lower surface uniformity. This approximately 1 μm decrease in film thickness over the 720 h-aging process indicates a lower deposition efficiency for the aged NPs compared to that of the non-aged NPs. This is primarily due to the altered charge state and NP–NP interactions of the Cu_2-x_S NPs, as demonstrated above by the morphological analysis of the film surfaces shown in Figure 2.

**Figure 4:**
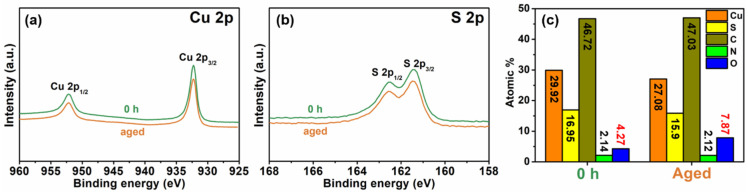
XPS spectra of the samples fabricated from the non-aged and aged NPs in (**a**) the Cu 2p region and (**b**) the S 2p region. A bar chart (**c**) showing the elemental compositions (at. %) of the samples fabricated from the non-aged and aged NPs is also provided.

To investigate the main driving force behind the change in the deposition behavior of the NPs with aging time, an XPS analysis was performed, and the results are presented in Figure 4 and Table S1. It was anticipated that differences in the chemical states of the non-aged and aged Cu_2-x_S NPs might provide clues to the aging effects. However, while the XPS spectra in the Cu 2p regions of the non-aged and 720 h-aged NPs in Figure 4a each exhibit two main peaks at approximately 932.3 eV (Cu 2p_1/2_) and 952.2 eV (Cu 2p_3/2_), no satellite peaks indicative of different valence states of Cu were observed [42]. The spectra of both the non-aged and aged NPs indicate the absence of Cu(II) and the presence of monovalent Cu as expected for Cu_2_S [26,43]. It was reported in the previous studies that copper-rich phases of Cu_2-x_S, such as roxbyite (Cu_1.81_S), djurleite (Cu_1.95_S), and chalcocite (Cu_2_S), yield XPS results similar to those of Cu_2_O in the Cu 2p region, which is dominated by the Cu(I) state [18,42,44,45,46]. Similarly, the XPS spectra in the S 2p region of both samples (Figure 4b) exhibit two major peaks at 161.5 eV and 162.5 eV due to S 2p_1/2_ and S 2p_3/2_, respectively, thus revealing the formation of Cu–S bonds [18,47]. These results indicate that the aging process does not lead to any significant changes in either the Cu valence state or chemical state of the Cu_2-x_S NPs.

Nevertheless, the bar chart of the atomic ratios of the two samples (Figure 4c) reveals a relatively high content of atomic oxygen in the aged NPs (7.87%) compared to the non-aged NPs (4.27%). This 3.6% increase in the oxygen content, along with an only 1.05% decrease in the S content, indicates an overall increase in oxidation going from the non-aged to aged sample. The XPS spectra in the O 1s region of non-aged and aged NP samples are shown in Figure S4. When comparing the main peaks of the two samples, different peak widths and intensities were observed, possibly due to the different bonding states of each sample (Figure S4a). The broad peak in O ls region collected from the aged NPs can be deconvoluted into two peaks at 532.0 eV and 531.2 eV (Figure S4c). The peak at 532.0 eV was assigned to the Cu–O–Cu bond (Cu_2_O), indicating that the surface of aged Cu_2-x_S was slightly oxidized as it was exposed to the atmosphere of air [48,49]. This peak was absent in the O 1s XPS spectra obtained from non-aged NPs (Figure S4b). The second peak of 531.2 eV, which appears in both samples, is caused by the C–O and C=O groups present on the surface of the NPs, produced as a by-product during the colloidal synthesis [50]. These XPS results indicate that the aging process led to formation of oxidized species, such as Cu_2_O on the Cu_2-X_S surface. In addition, the XRD patterns of the non-aged and aged NPs provided in Figure S5 were identical, thus confirming that slight oxidation during aging does not significantly alter the crystalline structure, even though the roxbyite phase of Cu_2-x_S contains Cu vacancy sites and is metastable at room temperature compared to djurleite or chalcocite [45,51]. Moreover, while oxidation is evident in the XPS results, the C- and N-containing OLAM ligands appear to be well maintained on the surface of the aged NPs, with only a minor (0.31%) increase in the content of atomic carbon and a 0.02% decrease in the content of atomic nitrogen in the aged sample, compared to a non-aged sample (Figure 4c). FTIR analysis was performed to demonstrate that the NP samples (aged and non-aged NPs) maintained the OLAM ligand, regardless of the aging process of colloidal NPs (Figure S6). The FTIR spectra of both samples clearly showed the bands within the range of 2840–3000 cm^−1^ attributed to the symmetrical and asymmetrical stretching modes of the CH_2_ and CH_3_, which are typical peaks from the OLAM component [52]. In addition, several peaks within the range of 1000–1500 cm^−1^ can be ascribed to the C–N and N–H stretching mode [53], which also indicates the presence of the OLAM. These FTIR features indicate that the surface of aged and non-aged Cu_2-x_S NPs are well-capped with the OLAM ligands. The XPS and FTIR results demonstrate that the change in the surface charge of the aged NPs, as reflected by the change in NP deposition behavior, is primarily due to surface oxidation rather than desorption of the ligands. In addition, the stronger interactions between the NPs in the solution might be strongly influenced by this aging effect, thus leading to the significant aggregation and the generation of a rough EPD film morphology.

Since the surface ligands of colloidal NPs play a major role in controlling the surface charge states, ligand engineering (i.e., the control of absorption or desorption states of the surface ligands) is generally applied to adjust the charge formation on colloidal NPs [54,55]. Moreover, the interactions between colloidal NPs are also stabilized by surface ligands in order to prevent aggregation. The ligand coverage on the surface of the NPs can be controlled via partial removal or addition of surface ligands during the precipitation steps [31]. Motivated by these ligand roles, a ligand engineering process was performed on the 720 h-aged NPs, in order to modulate the altered charge state due to the aging process and restore the particle-to-particle interactions. During this process, the excess ligands were absorbed onto the non-passivated NP surface to increase the surface ligand concentration and enable recovery of the aged NPs from severe aggregation.

**Figure 5:**
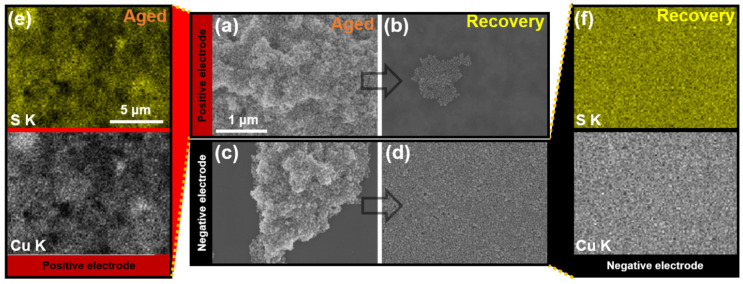
SEM images of the NP films fabricated on the positively charged substrate (top panel) and the negatively charged substrate (bottom panel) using the 720 h-aged NPs (**a**,**c**) and the recovered NPs (**b**,**d**). EDS mapping images of the aged sample on the positively charged substrate (**e**) and the recovered sample on the negatively charged substrate (**f**) are also shown.

The SEM images in Figure 5 compare the surface morphologies of NP films obtained from the unmodified 720 h-aged NPs and from the recovered NPs. Here, a change in the charge states of the NPs via the recovery process was clearly revealed, as was a change in the surface morphologies of the corresponding NP films. The use of the unmodified 720 h-aged NPs generated a rough NP film on the positively charged substrate (Figure 5a), while rough NP domains were observed on the negatively charged substrate (Figure 5c). By contrast, the recovered NPs produced a highly uniform film on the negatively charged substrate (Figure 5d), while only micrometer-sized NP domains were observed on the positively charged substrate (Figure 5b), along with stains which might be due to the presence of surplus ligands. The contrasting results of EPD film deposition using the aged NPs with and without ligand engineering clearly demonstrate that the net-charge on the recovered NPs is opposite to that on the aged (oxidized) NPs. Moreover, the modulation of the surface charge state of the colloidal NPs via ligand engineering plays a more dominant role than does the effect of NP aging. This charge control process is, therefore, an efficient tool for implementing selective electrode deposition during the EPD process. The significantly improved particle–particle interaction and assembly of the aged NPs to generate a highly uniform and dense film on the negatively charged substrate after ligand engineering is further demonstrated by comparison of the EDS mapping image in Figure 5f with that of the unmodified aged NPs on the positively charged substrate in Figure 5e.

**Figure 6:**
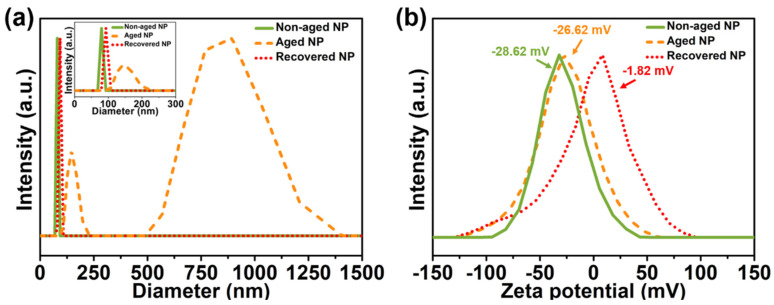
(**a**) Size distribution curves from dynamic light scattering (DLS) analysis for the NP samples (inset: magnified size distribution curves image for non-aged and recovered NPs) and (**b**) zeta potential distributions of NP samples dispersed in hexane; non-aged NPs, aged NPs, and recovered NPs are represented by the green solid, orange dash, and red dotted lines, respectively.

The aggregation state of several NP samples (non-aged, aged, and recovered NPs) in a non-polar solution was confirmed by analyzing the particle size through DLS. Intensity distributions of particle sizes collected from non-aged, aged, and recovered NPs are shown in Figure 6a. Since the diameter of the colloidal NPs measured through DLS indicates the hydrodynamic size including surface ligands [56], it may be generally larger than the particle size derived by TEM analysis. The average particle size of a non-aged NP sample is 74.6 nm with a narrow size distribution, which indicates that the NPs were well dispersed in hexane without NP aggregation. However, two peaks (average particle size of about 150 nm and 900 nm) with a broad size distribution were detected from the aged NP sample, revealing the existence of agglomerates with various diameters. As shown in the SEM image of the NP film fabricated from these aged NPs (Figure 5a,c), the presence of aggregates impairs the surface uniformity of the NP film. After the recovery process targeting these aged NPs, the average particle size collected from the samples represents 93 nm with a narrow size distribution, similar to that measured from non-aged NP sample (insert image in Figure 6a). These results confirm that the recovery process works effectively for the aged NP sample where aggregation has occurred, restoring the particle-to-particle interactions and allowing for disaggregation of NPs. The high dispersibility secured through the recovery process served as an important factor in producing NP film with high uniformity from the recovered NPs through the EPD process, as shown in Figure 5d. Compared to non-aged NPs, modulated surface charge states of aged and recovered NPs can be represented by the changes of zeta potential, which acts as a key factor in defining the moving direction and mobility of particles during the EPD process [15]. Based on the EPD results shown in the SEM images, the charge state of Cu_2-x_S NPs may become slightly more positive due to the aging effect. Zeta potential of the aged NP sample (−26.62 mV) is slightly more positive compared to that of the non-aged NP sample (−28.62 mV), which can support the phenomenon associated with the altered deposition behavior of aged NPs. In addition, the EPD results revealed that the net-charge on the recovered NPs is opposite to that on the aged NPs (Figure 5). Compared to the zeta potential of the aged NP, zeta potential of the recovered NPs shifted to significantly more positive (−1.82 mV), indicating that the NP’s charge state may be modulated from negative to positive through the ligand engineering process (Figure 6b). These results are consistent with EPD results of aged and recovered NP samples obtained by analyzing the NP films fabricated on the positive or negatively charged substrates.

**Scheme 1:**
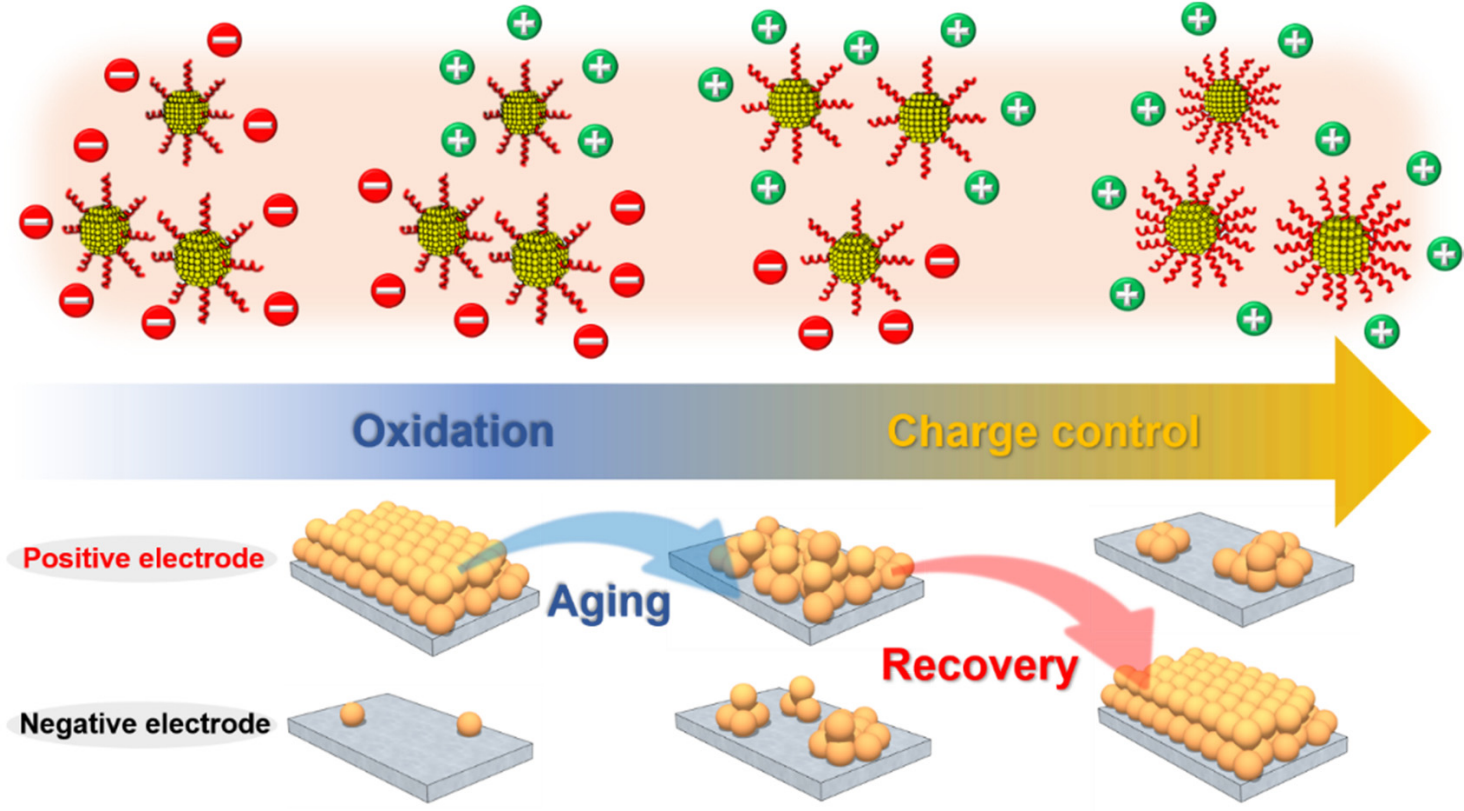
A schematic diagram showing the change in charge states and film morphologies of the colloidal Cu_2-x_S NPs depending upon the aging and recovery process.

As summarized schematically in Scheme 1, the difference in deposition behavior of the aged Cu_2-x_S NPs during the EPD process due to the inclusion or omission of a recovery process demonstrates that the interparticle interactions and surface charge formations are predominantly controlled by the ligands rather than the oxidation of NPs.

## 4. Conclusions

Two key electrophoretic deposition (EPD) parameters, namely the aging process of Cu_2-x_S NPs and the control of charge formation via surface ligand engineering, were revealed by analysis of the film morphologies obtained from variously aged NPs. The aging process was shown to result in different NP deposition behaviors by altering the charge states of oxidized NPs in the initial stage of aging. The aging state of the NPs was shown to be a key parameter in determining the direction of particle movement and the type of interparticle interactions during EPD. Moreover, a comparison of the NP films fabricated from aged NPs with and without ligand engineering process and zeta potential distributions collected from aged and recovered samples revealed that the surface ligands play an even more significant role in the charge formation than does the oxidation of the NPs due to aging. In conclusion, the ligand engineering of colloidal NPs is a key component of the EPD process for controlling charge formation and generating a uniform NP film from aged NPs via restoration.

## Data Availability

The data presented in this study are available on request from the corresponding author.

## Supplementary Materials

The following are available online at https://www.mdpi.com/2079-4991/11/1/133/s1, Figure S1: SEM images of the films deposited on the positively charged substrates using Cu_2-x_S NPs that were subjected to various aging times NPs. Figure S2: SEM images of the films deposited on negatively charged substrates using Cu_2-x_S NPs that were subjected to various aging times. Figure S3: Cross-sectional SEM images of the films deposited on positively charged substrates using Cu_2-x_S NPs that were subjected to various aging times. Figure S4: (a) O 1s XPS spectra of non-aged and aged NPs. The peak deconvolution of the O (1 s) XPS core level of (b) non-aged and (c) aged NPs. Figure S5: The XRD pattern of the 720-h aged NPs. The orange bars below the XRD pattern correspond to the reference of roxbyite phase (JCPDS #23-0958). Figure S6: FT-IR spectra of non-aged (green curve) and aged NPs (orange curve). Table S1: Elemental composition (at. %) based on the XPS analysis of the non-aged and aged Cu_2-x_S NPs.
