# Supplementary material for: Electrophoretic Deposition of Aged and Charge Controlled Colloidal Copper Sulfide Nanoparticles
Source: Nanomaterials (Basel). 2021 Jan 8;11(1):133. doi: 10.3390/nano11010133 (PMC7827911; doi:10.3390/nano11010133)
Supplement: Supplementary file 1 [file nanomaterials-11-00133-s001.pdf]

## Supporting Information

# Electrophoretic Deposition of Aged and Charge Controlled Colloidal Copper Sulfide Nanoparticles

**Yoonsu Park, Hyeri Kang, Wooseok Jeong, Hyungbin Son* and Don-Hyung Ha***

School of Integrative Engineering, Chung-Ang University, 84 Heukseok-ro, Dongjak-gu, Seoul 06974, Republic of Korea

* Correspondence: being@cau.ac.kr and dhha@cau.ac.kr

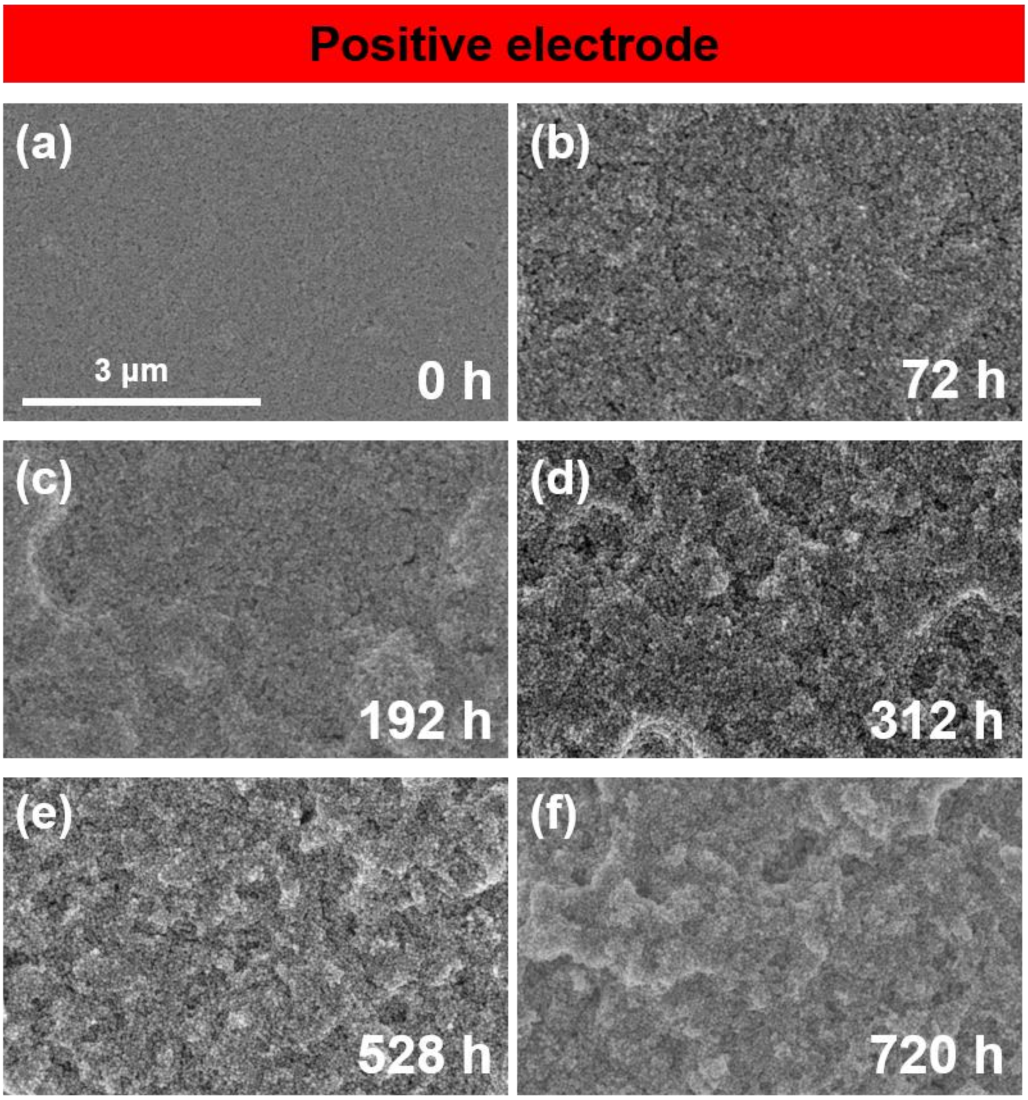


**Figure S1.** SEM images of the films deposited on the positively charged substrates using $Cu_{2-x}S$ NPs that were subjected to various aging times NPs.

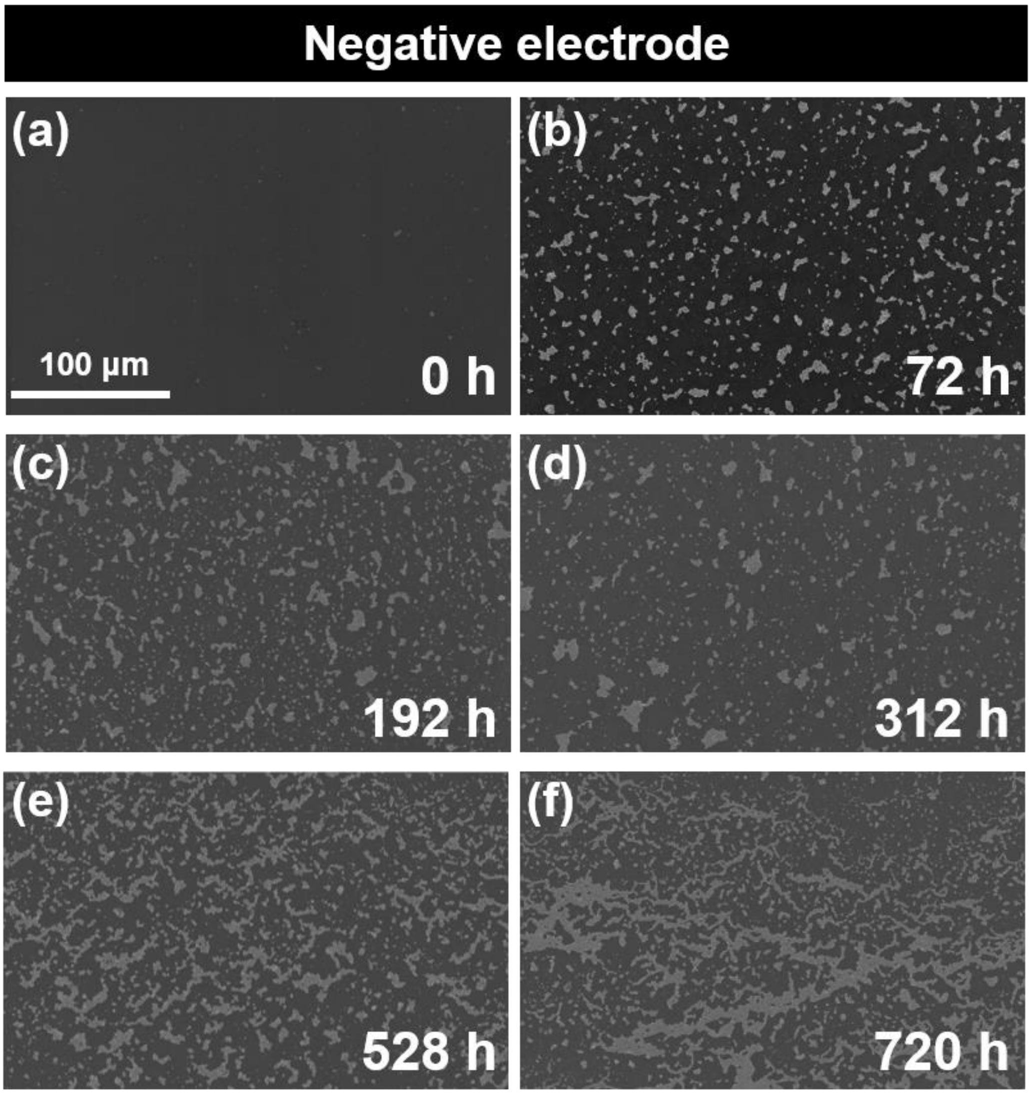


**Figure S2.** SEM images of the films deposited on negatively charged substrates using $Cu_{2-x}S$ NPs that were subjected to various aging times

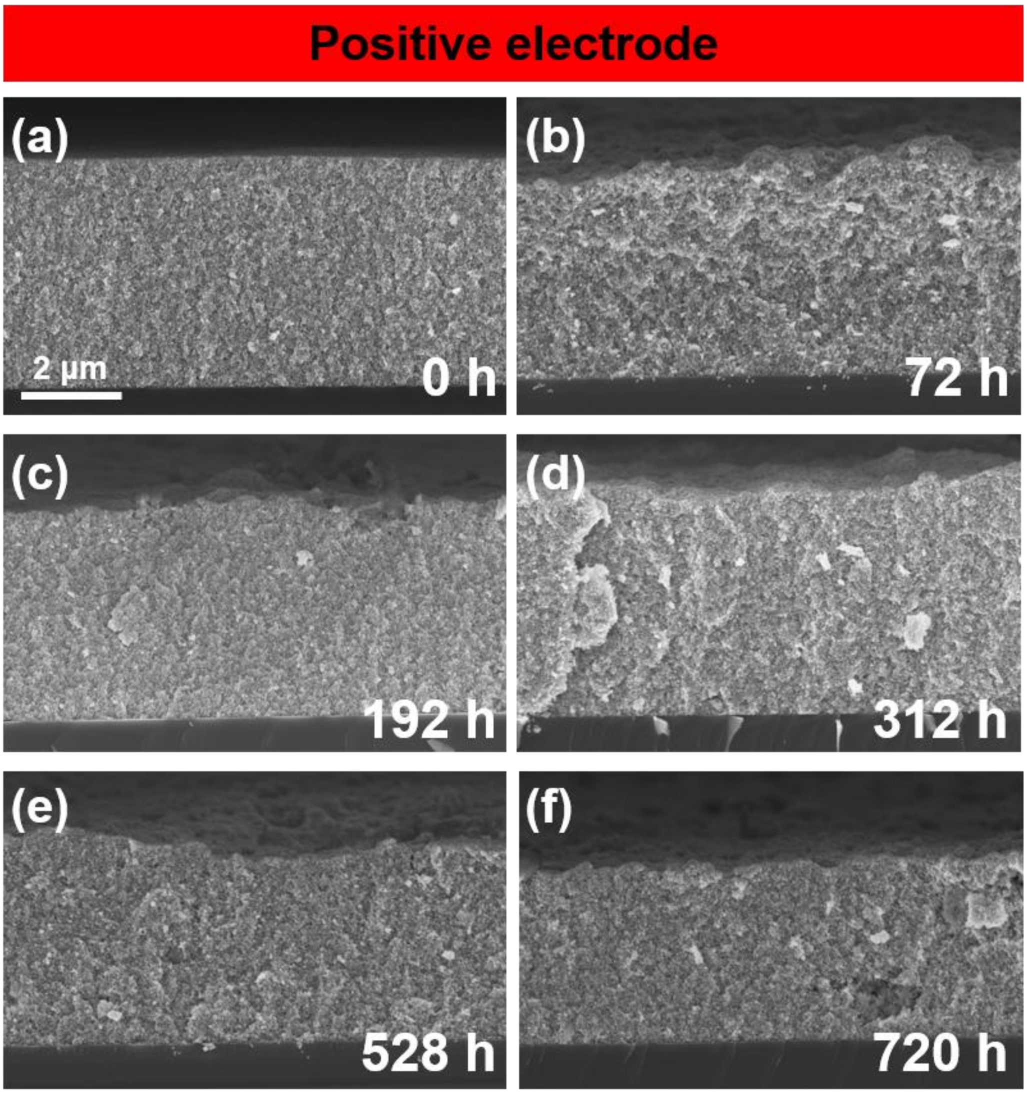


**Figure S3.** Cross-sectional SEM images of the films deposited on positively charged substrates using $Cu_{2-x}S$ NPs that were subjected to various aging times.

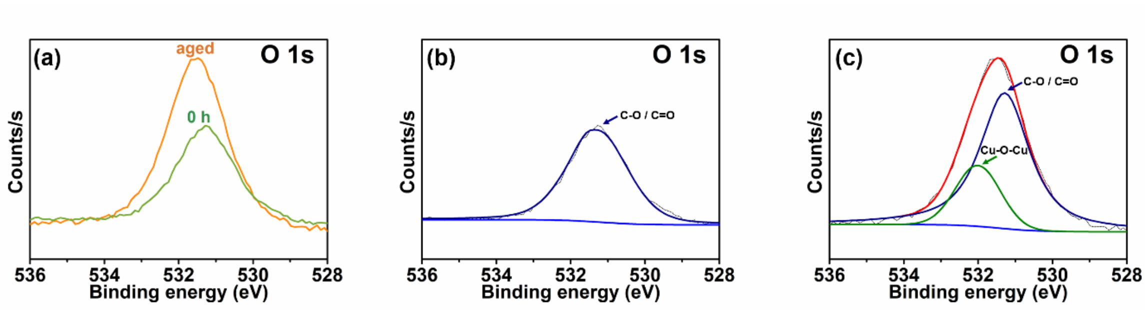


**Figure S4.** (a) O 1s XPS spectra of non-aged and aged NPs. The peak deconvolution of the O (1 s) XPS core level of (b) non-aged and (c) aged NPs.

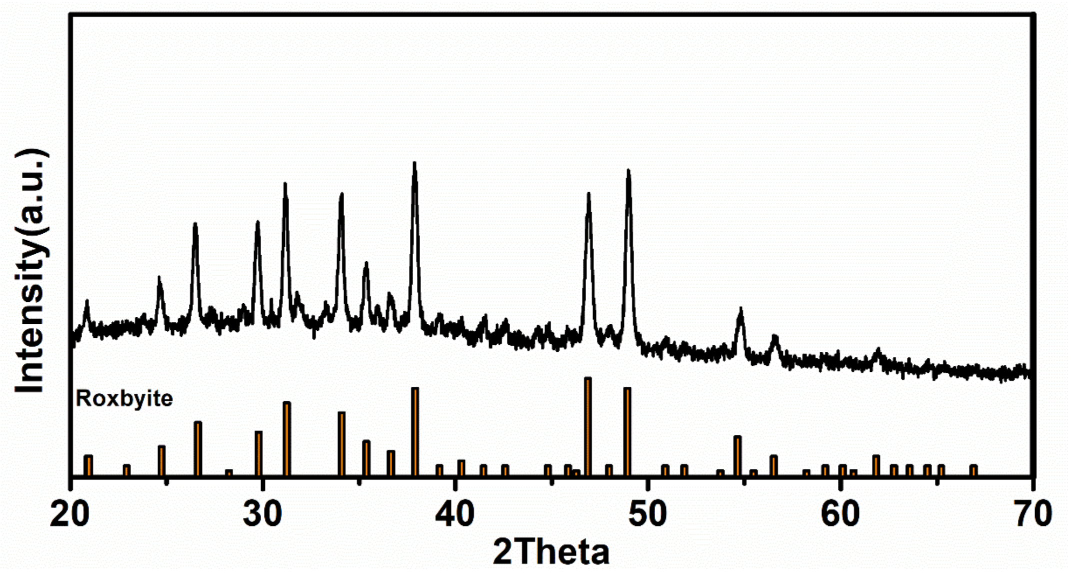


**Figure S5.** The XRD pattern of the 720-h aged NPs. The orange bars below the XRD pattern correspond to the reference of roxbyite phase (JCPDS #23-0958).

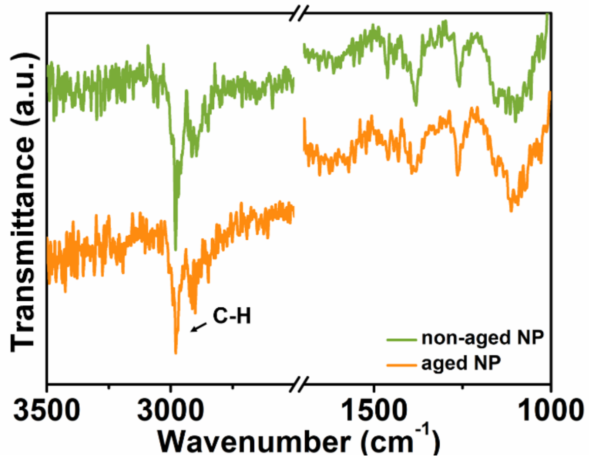


**Figure S6.** FT-IR spectra of non-aged (green curve) and aged NPs (orange curve).

**Table S1.** Elemental composition (at. %) based on the XPS analysis of the non-aged and aged $Cu_{2-x}S$ NPs.

| **Atomic %** | **Non-aged NP** | **Aged NP** |
|---|---|---|
| **C 1s** | **46.72** | **47.03** |
| **Cu 2p3** | **29.92** | **27.08** |
| **S 2p** | **16.95** | **15.9** |
| **N 1s** | **2.14** | **2.12** |
| **O 1s** | **4.27** | **7.87** |
